# Rare Co-occurrence of Ocular Myasthenia Gravis and Thyroid-Associated Orbitopathy (Ophthalmopathy) in an Individual With Hypothyroidism

**DOI:** 10.3389/fendo.2018.00801

**Published:** 2019-02-12

**Authors:** Ran An, Yan Li, Baiyuan Yang, Hui Wang, Yanming Xu

**Affiliations:** ^1^Department of Neurology, West China Hospital, Sichuan University, Chengdu, China; ^2^Department of Neurology, Chengdu Seventh People's Hospital, Chengdu, China

**Keywords:** myasthenia gravis, thyroid-associated orbitopathy, thyroid-associated ophthalmopathy, TAO, hypothyroidism, radioactive iodine therapy, hyperthyroidism, Graves' disease

## Abstract

Ocular myasthenia gravis (Ocular MG, OMG) shares many clinical features with thyroid-associated orbitopathy or thyroid-associated ophthalmopathy (TAO). In the rare instance of their coexistence, clinicians may fail to diagnose ocular MG when TAO is also present. Here we report the case of a patient with both TAO and ocular MG, whose “hyperthyroidism”—most likely the hashitoxicosis variant of Hashimoto's thyroiditis—rapidly transformed to hypothyroidism after radioactive iodine therapy. This is reminiscent of a previous case of a patient with MG, in whom disease onset coincided with the methimazole-induced transformation from hyper- to hypothyroidism. It is possible that the same transformation from “hyper-” to hypothyroidism, which occurred after radioactive iodine therapy and was accompanied by hypothyroidism-associated orbitopathy (ophthalmopathy), may have induced the development of myasthenia gravis in our patient. The hypothyroidism may have been caused by the radioactive iodine therapy and/or it may simply reflect the natural course of the hashitoxicosis variant of Hashimoto's thyroiditis. The co-occurrence of hypothyroidism, hypothyroidism-associated orbitopathy (ophthalmopathy) and ocular MG has never been reported. Our case highlights the need for clinicians to focus on overlapping symptoms of hyperthyroidism and the hashitoxicosis variant of Hashimoto's thyroiditis, and to carefully differentiate between them, especially when deciding on radioactive iodine therapy. In addition, our case highlights that the possible co-occurrence of TAO should be considered when a patient with thyroid disease displays both ptosis and eye movement dysfunction, and when only the ptosis is dramatically resolved after treatment with pyridostigmine bromide.

## Background

Autoimmune diseases, which result from specific immune responses against self structures, include autoimmune thyroid diseases and myasthenia gravis (MG). In autoimmune thyroid diseases, which include Hashimoto's thyroiditis and Graves's disease (GD), the body mounts immune responses against thyroid antigens (1). In myasthenia gravis, the body usually produces antibodies targeting acetylcholine receptors (AChRs) (1, 2), leading to defective nerve impulse transmissions to muscles and ultimately causing muscle weakness and abnormal susceptibility to fatigue. Autoimmune thyroid diseases and MG show many commonalities.

Ocular myasthenia gravis (Ocular MG, OMG) shares many clinical features with thyroid-associated orbitopathy or thyroid-associated ophthalmopathy (TAO) and is therefore difficult to diagnose when TAO is also present (3, 4). TAO can occur in patients with primary hypothyroidism, though it is more often reported in Graves' thyrotoxicosis. Here we report the case of a patient with TAO and ocular MG who underwent a rapid transformation from “hyper-” to hypothyroidism after radioactive iodine therapy.

## Case Report

A 35-year-old Chinese man, employed at a bank, showed the following abnormal thyroid function results during a health examination at our hospital in November 2016: thyroid-stimulating hormone (TSH), < 0.005 mU/L (normal, 0.27–4.2); free triiodothyronine (FT3), 26.11 pmol/L (3.6–7.5); free thyroxine (FT4), 59.16 pmol/L (12.0–22.0); anti-thyroid peroxidase antibodies (TPO-Ab), >600 IU/ml (<34); and anti-thyroglobulin antibodies (TG-Ab), >4,000 IU/ml (<115).

The same man was admitted to a local hospital in March 2017 for further evaluation. He reported palpitations, sweating, heat intolerance, weakness, fatigue, polyphagia, tremors, and increased defecation lasting throughout the previous 6 months. A physical examination revealed no distinctive abnormalities except for a goiter. The results of thyroid function tests were as follows: TSH, < 0.0004 mIU/L (normal, 0.35-4.94); FT3, 17.74 pmol/L (2.63-5.70); FT4, 33.64 pmol/L (9.01-19.05); TPO-Ab, >400 IU/ml (<30); TG-Ab, >2,000 IU/ml (<75); and anti-thyroid-stimulating hormone receptor antibodies (TSHR-Ab), 38.89 IU/L (<1.22). Thyroid ultrasonography revealed an uneven echoic involvement of the parenchyma, with iso-echo nodules of regular shape and a clear boundary in the right lobe and isthmus. The 24-h rate of radioactive iodine uptake increased, with a peak appearing in advance. The patient was diagnosed with “hyperthyroidism” and given the anti-thyroid drug Tapazole orally (10 mg, three times daily). After treatment for 20 days, the patient complained of itchy skin and a red rash. This was interpreted as an allergic reaction, so Tapazole was discontinued, radioactive iodine therapy was then given, and the patient was discharged.

**Figure 1:**
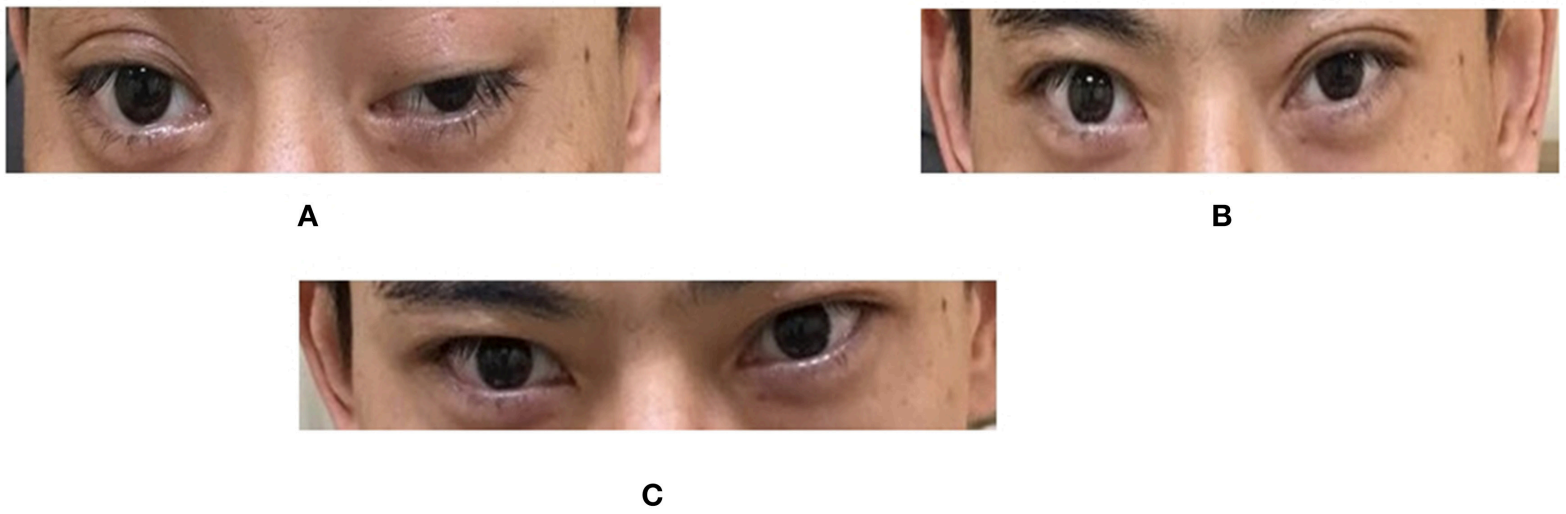
Eyelid symptoms before and after the neostigmine test. **(A)** Before the test. Both eyes, especially the left one, showed ptosis. **(B)** 30 min after the test. Ptosis improved dramatically, to a greater extent in the left eye. **(C)** 60 min after the test. Both eyelids recovered nearly to a normal state. Relative score = 100% (positive). In the neostigmine test, the most severely affected muscle group was chosen, the muscle power at baseline was scored, then neostigmine (1.5 mg) and atropine (0.5 mg) were administered intramuscularly. The patient was then evaluated every 10 min for a total of 60 min. Relative Score = (score at baseline − score when improvement was greatest after injection) / (score at baseline) × 100%. Relative scores ≤ 25% were classified as negative; >25% and < 60%, suspected positive; and ≥60%, positive.

In May 2017, the patient displayed ptosis of the left eye, which grew worse by the end of the day or after exertion, and which improved upon rest. He also exhibited diplopia and limited eye movement in all directions, which at its worst meant that he could not move his eyes at all. In addition, the patient reported generalized muscle ache and weakness. Thyroid function tests at the local hospital gave the following results: TSH, < 47.8642 mIU/L; FT3, <1.54 pmol/L; FT4, <5.15 pmol/L; TPO-Ab, >400 IU/ml; and TG-Ab, >2,000 IU/ml. The patient was diagnosed with hypothyroidism and took levothyroxine (L-T4, 75 mg per day) replacement therapy. Two weeks later, the symptoms of fatigue, muscle weakness and myalgia had completely disappeared. However, after 2 months of L-T4 therapy, ocular symptoms persisted, and the patient was admitted to the neurology department in the same local hospital in July 2017. A physical and neurological examination found no abnormalities except for ptosis of the left eye and the limited movement of both eyes in all directions, without proptosis, periorbital or limb edema. TSH, FT3, and FT4 were normal. TPO-Ab was >400 IU/ml, TG-Ab was 1416.67 IU/ml, and serum lactic acid was 2.4 mmol/L (normal, 0.5–2.2). Complete blood count, tests of liver and kidney function, as well as levels of creatine kinase and serum tumor markers were normal. Magnetic resonance imaging of the brain, cervical vertebra, and the orbital cavity was normal, as were the electrocardiogram and echocardiography. Contrast-enhanced computed tomography of the chest was normal, revealing no obvious thymic hyperplasia or thymoma. Thyroid ultrasonography revealed uneven echoic involvement of the parenchyma, with iso-echo nodules in the right lobe. Low- and high-frequency repetitive nerve stimulation did not reveal abnormal decrease or increase in the amplitude of compound muscle action potential. The Ptosis was apparently alleviated, based on the positive response in the neostigmine test (Figure 1) (5, 6), but restricted eye movement and diplopia remained. Conventional enzyme-linked immunosorbent assays (ELISAs) showed normal levels of antibodies against AChRs (0.39 nmol/L; normal, <0.4), muscle-specific kinase (<0.4 U/ml; normal, < 0.4), skeletal muscle, myocardium, titin, and SOX1. Ocular MG was suspected, and the patient was prescribed pyridostigmine bromide (60 mg, three times a day) along with the previous L-T4 regimen.

In August 2017, the patient was admitted to our neurology department complaining of restricted eye movements. The following test results were obtained upon admission: serum lactic acid, 2.7 mmol/L (normal, 0.7-2.1); TSH, FT3, and FT4, normal; TPO-Ab, >600 IU/ml; and TG-Ab, >4,000 IU/ml. Normal findings were obtained for complete blood count, erythrocyte sedimentation rate, liver and kidney function, electrolytes, serum glucose, serum lipid, creatine kinase, and serum tumor markers. A thyroid ultrasonography revealed uneven hypoechoic involvement of the parenchyma, with mild hyper-echo nodules in the right lobe. Low- and high-frequency repetitive nerve stimulation did not reveal abnormal changes in the amplitude of compound muscle action potential. Nerve conduction tests and needle electromyography both provided normal findings. Contrast-enhanced magnetic resonance imaging of the orbital cavity revealed thickened extraocular muscles in both eyes, with an obvious uneven enhancement.

**Figure 2:**
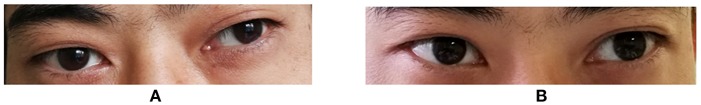
Eyelid symptoms after cessation of pyridostigmine bromide therapy. **(A)** At 1 month after cessation of therapy (Dec. 2017). **(B)** At 3 months after cessation of therapy (Feb. 2018). At both time points, the left eyelid was nearly unaffected, showing only mild abnormality.

Given the possibility of a mitochondrial disease, such as chronic progressive external ophthalmoplegia, a muscle biopsy was conducted, and findings did not indicate any distinctive abnormalities. The patient was started on combination therapy of L-T4 and pyridostigmine bromide, which led to a stable condition of limited eye movement and mild diplopia. He stopped using pyridostigmine bromide in November 2017 when he began planning for a baby. A follow-up in December 2017 and February 2018 showed only mild abnormality in the left eyelid (Figure 2).

Table 1 summarizes the results of the thyroid function tests from the beginning in November 2016 to last follow-up. During the follow-up, radioimmunoprecipitation assays revealed the presence of antibodies against AChRs (7.310 nmol/L; normal, <0.5) and muscle-specific kinase (0.005 nmol/L; normal, < 0.05). Later on, titer of anti-AChR antibodies was 0.678 nmol/L (normal, <0.4) based on an ELISA test and 1.864 nmol/L (normal, <0.5) based on a radioimmunoprecipitation assay (Table 2).

**Table 1:** Changes in thyroid function results from the beginning to the last follow-up.

**No**	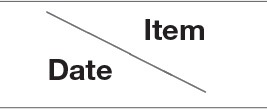	**TSH**	**FT3**	**FT4**	**TPO-Ab**	**TG-Ab**	**TSHR-Ab**
1	Nov. 2016	< 0.005↓ (0.27–4.2 mU/L)	26.11↑ (3.6–7.5 pmol/L)	59.16↑ (12.0–22.0 pmol/L)	>600↑ (< 34 IU/ml)	>4000↑ (< 115 IU/ml)	ND
2	Feb. 2017	0.0009↓ (0.35–4.94 mIU/L)	17.96↑ (2.63–5.70 pmol/L)	41.36↑ (9.01–19.05 pmol/L)	>400↑ (< 30 IU/ml)	1823.86↑ (< 75 IU/ml)	35.11↑ (< 1.22 IU/L)	
3	Mar. 2017	0.0004↓	17.74↑	33.64↑	>400↑	>2000↑	38.89↑
4	May 2017	47.8642↑	< 1.54↓	< 5.15↓	>400↑	>2000↑	ND
5	Jun. 2017	2.4451	4.05	13.04	ND	ND	>40↑
6	Jul. 2017	1.8375	3.65	13.63	>400↑	1416.67↑	ND
7	Aug. 2017	0.512	4.28	16.97	>600↑	>4000↑	ND
8	Dec. 2017	0.606	4.55	16.39	>600↑	>4000↑	22.28↑ (< 3 IU/L)

*FT3, free triiodothyronine; FT4, free thyroxine; ND, not done; TG-Ab, thyroglobulin antibodies; TPO-Ab, thyroid peroxidase antibodies; TSH, Thyroid-stimulating hormone; TSHR-Ab, thyroid-stimulating hormone receptor antibodies. Tests in rows 1, 7, and 8 were performed at our hospital; others were performed at another hospital. In March 2017, radioactive iodine therapy was performed. In May 2017, ocular myasthenia gravis and thyroid-associated orbitopathy occurred, and hypothyroidism was detected*.

**Table 2:** Titers of anti-AChR and anti-MuSK antibodies assayed by ELISA or radioimmunoprecipitation assay.

	**ELISA**		**Radioimmunoprecipitation**	
**Date**	**Anti-AchR (< 0.4 nmol/L)**	**Anti-MuSK (< 0.4 U/ml)**	**Anti-AchR (< 0.5 nmol/L)**	**Anti-MuSK (≤0.05 nmol/L)**
Jul. 2017	0.39	< 0.4	ND	ND
10 Dec. 2017	ND	ND	7.310	0.005
20 Dec. 2017	0.678	ND	1.864	ND

*ELISA, enzyme-linked immunosorbent assay; AChR, acetylcholine receptor; MuSK, muscle-specific kinase; ND, not done. Normal ranges are indicated below the antibody descriptions*.

## Discussion

After the patient's first admission to a local hospital in March 2017, he was diagnosed with Graves's disease on the basis of abnormal thyroid function results with hyperthyroidism, increased titers of TPO-Ab, TG-Ab, and TSHR-Ab, and an increased 24-h rate of radioactive iodine uptake. However, a review of the initial ultrasound images of the thyroid gland and repeated ultrasonography which revealed uneven (nodular) hypoechoic areas, raises the possibility of the hashitoxicosis variant of Hashimoto's thyroiditis at the initial presentation rather than Grave's disease, which usually displays on ultrasound images as diffused hypoechogenicity without nodules (4). Unfortunately, because the fine needle puncture of the thyroid was not performed routinely as the first choice for the diagnosis of the thyroid disease, the biopsy and pathological examination of the thyroid gland was not performed, which could have aided in a correct diagnosis at the initial presentation.

The differential diagnosis of hyperthyroidism should include Grave's disease and the hashitoxicosis variant of Hashimoto's thyroiditis. The latter may show a hyperthyroid phase virtually indistinguishable from Graves's disease, including the elevated thyroid uptake of radioactive iodine and the presence of thyroid-stimulating immunoglobulins. However, hyperthyroidism is transient in the hashitoxicosis variant of Hashimoto's thyroiditis, and after 3–24 months evolves into permanent hypothyroidism (7). The hypothyroidism in our patient may have been caused by radioactive iodine therapy and/or the natural progression of the hashitoxicosis variant of Hashimoto's thyroiditis. However, if the suspected diagnosis of the hashitoxicosis variant of Hashimoto's thyroiditis was true in our patient, treatment with radioactive iodine therapy may have been inappropriate, because this can further destroy thyroid tissue and exacerbate the symptoms of hypothyroidism. The present case highlights the need for clinicians to carefully differentiate between the hashitoxicosis variant of Hashimoto's thyroiditis and Graves's hyperthyroidism, in light of their overlapping symptoms and the damage that may result from inappropriate application of radioactive iodine therapy. If necessary, the thyroid should be biopsied and analyzed by histopathology in order to ensure accurate, complete diagnosis.

Our patient showed elevated serum concentrations of anti-AChR antibodies (Table 2), consistent with previous reports (8). This observation, together with the positive neostigmine test (Figure 1) and substantial improvement in ptosis following the first treatment with pyridostigmine bromide, permitted a definite diagnosis of MG. This highlights the usefulness of repeat testing for anti-AChR antibodies if initial results are normal.

In addition to hypothyroidism and ocular symptoms, our patient showed generalized muscle ache and weakness on May 2017, which disappeared after 2 weeks of levothyroxine therapy. Muscle ache and weakness are considered characteristics of hypothyroid myopathy (8). Although most patients with hypothyroidism with MG give a negative result on the edrophonium test (8), while our patient tested positive on a neostigmine test. It is likely that the myopathic signs and symptoms of the patients in that previous study, disappeared with thyroid hormone replacement therapy, in turn suggesting that most myasthenia gravis symptoms in those patients were manifestations of hypothyroid myopathy, and that such symptoms can disappear with thyroid hormone replacement therapy, without the need for anti-myasthenic medication. However, our patient showed ptosis that did not respond to levothyroxin therapy. We concluded that the ptosis was a clinical manifestation of myasthenia gravis and independent of hypothyroid myopathy. Consistent with this idea, the ptosis nearly disappeared after a few months of treatment with pyridostigmine bromide.

Initially, our patient required anti-myasthenic medications to relieve ptosis. Subsequently, even at 1 or 3 months after cessation of pyridostigmine therapy, the left eyelid showed only mild abnormality (Figure 2). This leads us to suggest that the thyroid disease and MG were distinct yet interacting conditions in our patient. Controlling the hypothyroidism in our patient may also have alleviated the MG symptoms to some extent. The mild ocular abnormality in our patient, even after withdrawal of pyridostigmine bromide, is consistent with the observation that MG is often milder and more likely to show ocular involvement in patients with autoimmune thyroid disease, than in patients with non-autoimmune thyroid disease or no thyroid disease etc. (3).

Our patient displayed restricted eye movements and thickened extraocular muscles of both eyes, which are typical of TAO. The presence of ptosis in a patient with TAO should alert the clinician to the possible presence of MG, since the clinical features of TAO and ocular MG overlap substantially, and ptosis has been described as a rare feature of TAO (3). Indeed, the ptosis in our patient appears to have been a symptom of MG, further identifying the existence of OMG in the context of TAO. Our case highlights that the possible co-occurrence of TAO should be considered if a patient with thyroid disease displays both ptosis and eye movement dysfunction, and if only the ptosis dramatically resolves after treatment with pyridostigmine bromide. If necessary, magnetic resonance imaging of the orbit can be performed to guide diagnosis and further therapy.

TAO may occur more frequently among Graves' disease patients with ocular MG, than among Graves' disease patients without MG or with generalized MG. Euthyroid ophthalmopathy occurs more frequently among Graves' disease patients when they also have MG, suggesting a preferential association between the ocular manifestations of Graves' disease and MG (9). This previous study suggested that the co-occurrence of ocular MG and TAO may be due to a shared genetic background as well as immunological cross-reactivity against common autoimmune targets in the eye muscle. Our case is consistent with the preferential association between hypothyroidism-associated orbitopathy (ophthalmopathy) and ocular MG. Whole-exome sequencing of our patient and his parents did not identify any obvious gene mutations or polymorphisms in coding regions that may help explain an association between thyroid-associated ophthalmopathy and ocular MG (data not shown). Large genetic studies are needed to examine this potential association in detail.

Previous studies described a patient with MG in whom disease onset coincided with the methimazole-induced transformation from hyper- to hypothyroidism (10). It is possible that the transformation from “hyper-” to hypothyroidism in our patient is analogous, inducing the development of MG, although it occurred after radioactive iodine therapy and was accompanied by hypothyroidism-associated orbitopathy (ophthalmopathy). The hypothyroidism may have been caused by the radioactive iodine therapy and/or it may simply reflect the natural course of the hashitoxicosis variant of Hashimoto's thyroiditis. This appears to be the first report of the triple occurrence of hypothyroidism, hypothyroidism-associated orbitopathy (ophthalmopathy) and ocular MG. This case therefore deserves significant attention because of its implications in research and clinical practice.

## Concluding Remarks

MG is usually reported in hyperthyroidic patients, rarely in patients with hypothyroidism. We present here a rare case of co-occurrence of MG, hypothyroidism and TAO after radioactive iodine therapy for “hyperthyroidism.” This case highlights the need for clinicians to be aware of the possible co-occurrence of such comorbidities, which have implications for treatment and management. Clinicians should carefully differentiate between hyperthyroidism and the hashitoxicosis variant of Hashimoto's thyroiditis, especially when considering radioactive iodine therapy. Our case further highlights the need to consider the co-occurrence of TAO if pyridostigmine bromide only partly resolves ocular MG symptoms in patients with thyroid disease. If necessary, magnetic resonance imaging of the orbit can be performed to guide diagnosis and therapy.

## Ethics Statement

The study protocol was approved by the Ethics Committee of Sichuan University and written informed consent was obtained from participants.

## Author Contributions

RA, YL, BY, and HW collected patient data and wrote the manuscript. YX conducted the study and revised the manuscript.

### Conflict of Interest Statement

The authors declare that the research was conducted in the absence of any commercial or financial relationships that could be construed as a potential conflict of interest.
